# H-BIM and Artificial Intelligence: Classification of Architectural Heritage for Semi-Automatic Scan-to-BIM Reconstruction

**DOI:** 10.3390/s23052497

**Published:** 2023-02-23

**Authors:** Valeria Croce, Gabriella Caroti, Andrea Piemonte, Livio De Luca, Philippe Véron

**Affiliations:** 1 Department of Energy, Systems, Land and Construction Engineering (DESTEC), University of Pisa, 56122 Pisa, Italy; 2 Civil and Industrial Engineering, ASTRO Laboratory, University of Pisa, 56122 Pisa, Italy; 3 UMR MAP 3495 CNRS/MC, Campus CNRS Joseph-Aiguier, 13402 Marseille, France; 4 LISPEN EA 7515, Arts et Métiers Institute of Technology, 13100 Aix-en-Provence, France

**Keywords:** machine learning, BIM, H-BIM, artificial intelligence, point cloud, classification, digital heritage, cultural heritage, 3D models, scan-to-BIM, semantic segmentation

## Abstract

We propose a semi-automatic Scan-to-BIM reconstruction approach, making the most of Artificial Intelligence (AI) techniques, for the classification of digital architectural heritage data. Nowadays, Heritage- or Historic-Building Information Modeling (H-BIM) reconstruction from laser scanning or photogrammetric surveys is a manual, time-consuming, overly subjective process, but the emergence of AI techniques, applied to the realm of existing architectural heritage, is offering new ways to interpret, process and elaborate raw digital surveying data, as point clouds. The proposed methodological approach for higher-level automation in Scan-to-BIM reconstruction is threaded as follows: (i) semantic segmentation via Random Forest and import of annotated data in 3D modeling environment, broken down class by class; (ii) reconstruction of *template* geometries of classes of architectural elements; (iii) propagation of *template* reconstructed geometries to all elements belonging to a typological class. Visual Programming Languages (VPLs) and reference to architectural treatises are leveraged for the Scan-to-BIM reconstruction. The approach is tested on several significant heritage sites in the Tuscan territory, including charterhouses and museums. The results suggest the replicability of the approach to other case studies, built in different periods, with different construction techniques or under different states of conservation.

## 1. Introduction

In recent years, the Building Information Modeling (BIM) methodology was transferred from the realm of new construction to that of the built heritage. Since the early studies by Murphy and Dore [1,2], the scientific literature on Heritage or Historic-BIM (H-BIM) has expanded [3,4,5,6,7,8], aiming to illustrate how geometrical data can be linked to: architectural grammar and styles [9,10,11], material characterization [12], degradation patterns [13], façade interventions and historical layers [14,15], structural damage and FEM analysis [16,17,18], data collection and simulation of environmental parameters [19], archival photographs [20] and text documents [21,22]. 

Hichri et al. [8] and Macher et al. [23] emphasized that H-BIM techniques require the transition from the existing condition of the object to the modeling environment. The shift from the *as-built* condition (registration of a building after construction) to the *as-is* representation (registration of its current condition) implies reference to surveying data, as point clouds acquired via laser scanning or photogrammetry [24] and reverse engineering techniques. On the one hand, the elaboration of 3D surveying for the construction of BIM models, known as Scan-to-BIM [21], is seen as a manual, time-consuming and subjective process [3,5]; on the other hand, the emergence of Artificial Intelligence (AI) techniques in the architectural heritage domain [25,26,27] is reshaping the approach of heritage experts towards the interpretation, recognition and classification of building components on raw surveying information. Based on this consideration, this work proposes a semi-automated procedure to enable the construction of BIM models of heritage objects and sites, starting from 3D survey data that are classified via a supervised Machine Learning (ML) method.

The paper is structured as follows: Section 2 provides a literature review on Scan-to-BIM techniques and AI-based semantic segmentation processes. Section 3 presents case studies on which the proposed methodology was tested (*Materials*), and concurrently illustrates the different steps of the reconstruction approach (*Methods*). In Section 4 and Section 5, the results are presented and discussed, while Section 6 draws conclusions and future developments.

## 2. Related Work

### 2.1. State-of-the-Art Scan-to-BIM Reconstruction Processes

**Figure 1:**
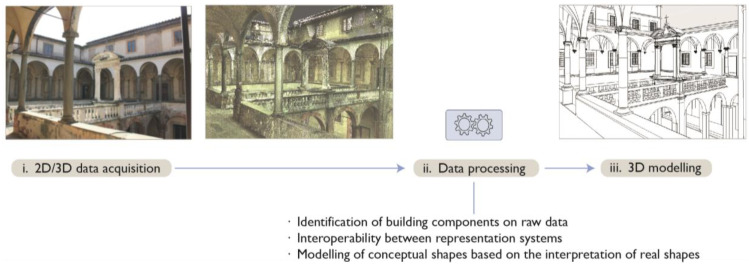
Steps of the Scan-to-BIM workflow using example from Grand-Ducal cloister, Pisa Charterhouse.

Scan-to-BIM processes [7,21,28] focus on translating existing survey data, as point clouds, into BIM. They involve three main steps: (i) data acquisition by laser scanning or photogrammetry; (ii) processing of survey data; and (iii) 3D modelling (Figure 1). In the processing phase (ii), it is essential to semantically describe the objects that make up a building over unstructured point clouds [28]. This interpretative issue is a major bottleneck in current research, as the main limits of the Scan-to-BIM processing workflow are identified as:

- Difficulties in modeling complex or irregular elements and representing architectural details of existing buildings [1,29,30], and the need to intervene with classification, hierarchical organization and simplification assumptions [14,23];
- Measurement uncertainties [23], as surveying data may contain occlusions [31];
- Compared to BIM for new constructions, there is an absence of pre-defined, extensive libraries of parametric objects [3] and lack of existing standards for H-BIM artefacts [1,28,30];
- High conversion effort [1], since most BIM software for new buildings offer tools for the construction of regular and standardized objects while the free-form geometry modeling functions that are available are limited [15,29,32,33].

For the above limits, Scan-to-BIM techniques are never unambiguous. However, they can be distinguished based on the degree of human involvement in the data processing stage, classified as manual (Section 2.1.1) or semi-automated (Section 2.1.2).

#### 2.1.1. Manual Scan-to-BIM Methods

**Figure 2:**
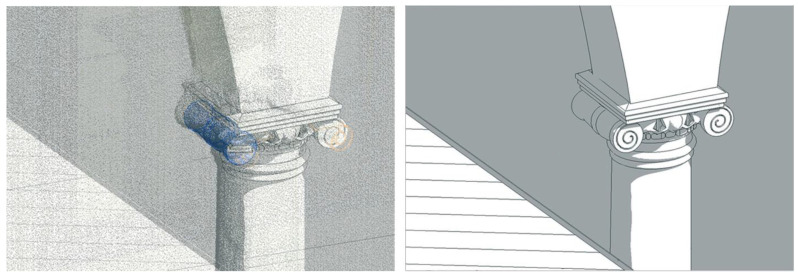
Instantiation of a capital by direct reconstruction over the raw point cloud using example from Grand-Ducal cloister, Pisa Charterhouse.

Most common approaches to the Scan-to-BIM are manual, as they require visual recognition and subsequent manual tracing of building components starting from a point cloud (Figure 2). Extensive literature reviews provided by Logothetis et al. [6], Volk et al. [7], Tang et al. [34] and more recently by López et al. [3] and Pocobelli et al. [4], demonstrate that manual methods [1,35], although widely consolidated, result in time-consuming, laborious processes. Indeed, operators are asked to manually identify, isolate and reconstruct each class of building elements [7,23]. This entails a considerable amount of time and resources, besides implying the risk of making too subjective choices [36].

#### 2.1.2. Semi-Automated Scan-to-BIM Methods

**Figure 3:**
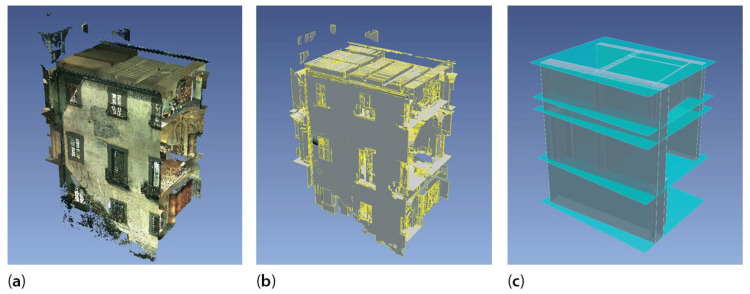
Primitive fitting of perimetral walls and slabs by EdgeWise Building. Raw point cloud (**a**); fit planes (**b**); BIM objects (**c**) using example from Grand-Ducal cloister, Pisa Charterhouse [46].

Fundamental issues in the definition of semi-automated methods are the recognition and labelling of data points on raw point clouds with a named object or object class (e.g., windows, columns, walls, roofs, etc.) [34,35,36,37,38]. Existing methods can be distinguished according to the solution identified over time for this issue: 

Primitive fitting methods. They fit simple geometries, such as planes, cylinders and spheres [39], to sets of points in the scene via robust estimation of the primitive parameters. The Random Sample and Consensus [40] and the Hough Transform [41] are common algorithms of this type, used in commercial solutions for the semi-automatic recognition of walls, slabs and pipes, proposed by software houses [3,42,43,44] including: EdgeWise Building by ClearEdge3D (clearedge3d.com) as a complement for Autodesk Revit; Scan-to-BIM Revit plug-in by IMAGINiT Technologies (imaginit.com); and Buildings Pointfuse from Arithmetica (pointfuse.com). Primitive fitting methods mostly apply to indoor environments [31,37,38,42] for the detection of planar elements, as floors and walls [23,37,45]. Shape extraction and BIM conversion are limited to simple geometries with standardized dimensions; application to complex existing architectural structures, varying in forms and types, is hardly possible unless the model is oversimplified (Figure 3) [23,42].

Mesh-reconstruction methods. For each architectural component or group thereof, a mesh is reconstructed via triangulation techniques, starting from the distribution of points in the original point cloud. References [15,17,29,31,47,48,49] converted 3D textured meshes derived from surveying into BIM objects; however, the mesh manipulation and geometric modification are limited as the mesh models cannot be edited and controlled by parametric BIM modeling [29].

Reconstruction by shape grammar and object libraries. Such approaches rely on the construction of suitable 3D libraries of architectural elements (families) to handle the complexity of materials and components that characterizes historic architecture [10,50,51,52]. In detail, De Luca et al. [53] studied the formalization of architectural knowledge based on the analysis of architectural treatises, to generate template shape libraries of classical architecture. Murphy et al. [54] modelled interactive parametric objects based on manuscripts ranging from Vitruvius to Palladio to the architectural pattern books of the 18th century. Since relying on the formalization of architectural languages as derived from treatises of historical architecture, such methods are valid regardless of the modeling type or representation chosen [53]. 

Reconstruction by generative modelling. In this case, the reconstruction is again guided by the formalization of architectural knowledge, and VPLs are considered to manipulate each geometry by interactively programming, via a graphical coding language made up of nodes and wires, the set of modeling procedures, primitive adjustments and duplication operations performed in 3D space [33,53,55]. Grasshopper, a visual programming interface for Rhino3D, and Dynamo, a plug-in for Autodesk Revit, are commonly used for these tasks in the case of new constructions. By contrast, VPLs are rarely exploited for existing monuments and sites. The 3D content could be created, based on surveying data [17,48,55,56,57], by a series of graphic generation instructions, repeated rules and algorithms [58]. The release of Rhino.Inside.Revit (rhino3d.com/it/features/rhino-inside-revit, accessed on 18 December 2022), allowing Grasshopper to run inside BIM software as Autodesk Revit, goes in the direction of novel VPL-to-BIM connection tools.

### 2.2. State-of-the-Art AI-Based Semantic Segmentation

In the digital heritage field, ML and Deep Learning (DL) techniques emerge to help digital data interpretation, semantic structuring and enrichment of a studied object [25] e.g., to assist the identification of architectural components [59], the re-assembly of dismantled parts [60], the recognition of hidden or damaged wall regions [61], and the mapping of spatial and temporal distributions of historical phenomena [62].

**Figure 4:**
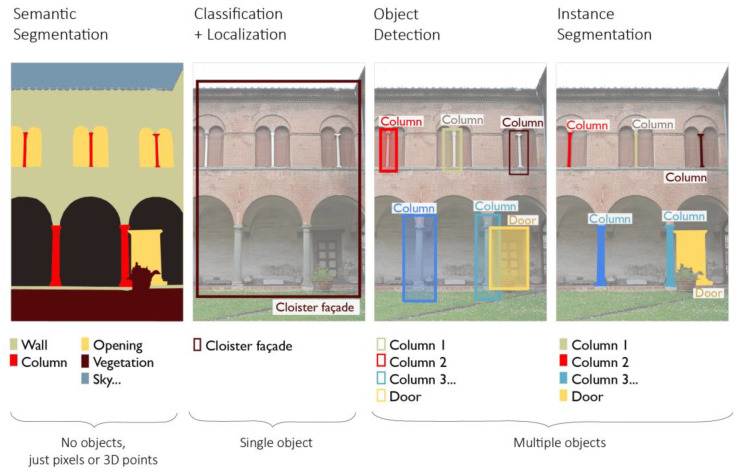
Semantic segmentation compared to other computer vision tasks using example from Cloister of the National Museum of San Matteo, Pisa.

In the architectural heritage domain, AI techniques have proven to be crucial in streamlining the so-called semantic segmentation process, understood as the reasoned subdivision of a building into its architectural components (e.g., roof, wall, window, molding, etc.), starting from surveying data. With respect to other common computer vision tasks exploiting AI, such as object recognition, instance localization and segmentation, the semantic segmentation process classifies pixels or points as belonging to a certain label and performs this operation for multiple objects of the 2D image or of the 3D unstructured scene (Figure 4). The term *semantic*, indeed, underlines that the breakdown is done by referring to prior knowledge on the studied 2D/3D architectural scenes.

Though earlier experiments of digital heritage classification were geared towards the semantic segmentation of images [61,63,64], research is now moving in the direction of segmenting textured polygonal meshes [27] and/or 3D point clouds [65]. In the architectural domain, the classification is either focused on automatically recognizing, via ML algorithms and through a suitable amount of training data, on the one hand, the presence of alterations on historical buildings [66] or the mapping of materials (*texture-based* approaches) [27,67,68], and, on the other hand, the distinction into architectural components based on prior historical knowledge (*geometry-based* approaches) [26,69,70,71].

Depending on the type of approach chosen, the classification can act on either two kinds of properties of the raw data: (a) geometric features, such as height, planarity, linearity, sphericity, etc. [72], that are better suited for the recognition of architectural components based on respective shapes of elements, or (b) colorimetric attributes, such as RGB, HSL or HSV color spaces [66], that are widely used for the identification of decay patterns (as biological patina or colonization, chromatic alterations, spots, etc.) or of materials.

Geometry-based classification techniques, formerly exploited for classifying urban scenes [17,72,73], are now applied to the scale of the individual building, for the segmentation of walls, moldings, vaults, columns, roofs, etc. [70]. Grilli et al. [70] investigated the effectiveness of covariance features [72] in training a Random Forest (RF) classifier [74] for architectural heritage, even demonstrating the existence of a correlation between such features and many main dimensions of architectural elements. 

### 2.3. Open Issues Arising from the State-of-the-Art Methods

The literature review in Section 2.1 proves that data segmentation and classification are essential steps in common Scan-to-BIM workflows, enabling:

- A breakdown of the survey data into subsets of elements (pixels or points) sharing the same features, whether geometric or radiometric, extracted from 2D or 3D descriptors and according to predefined criteria (segmentation);
- The assignment of a label to each subset (classification or semantic segmentation).

Although this process has been considered to be mostly manual and performed by a single operator, the evolution of recent research in the application of RF algorithms to the classification of digital heritage point clouds (see Section 2.2), suggests the possible automation of Scan-to-BIM reconstruction processes. The semantically segmented point cloud, in which different architectural elements are finally distinguished as an outcome of geometry-based classification techniques, could in fact be considered as a basis for the reconstruction of H-BIM models. To date, besides primitive shape fitting approaches [75], no research has considered the possible integration of semi-automatically annotated point clouds and H-BIM environments. This still-uncertain and unclear transition, deserving more in-depth analysis and worth the exploration of the operational challenges of a scan-to-BIM via ML model, constitutes the research line of the present work.

## 3. Materials and Methods

### 3.1. Materials

The semantic segmentation and Scan-to-BIM reconstruction methodology was tested on three point clouds of historic buildings in the Tuscany territory (Italy), acquired either by laser scanning or photogrammetry, alongside traditional topographic instruments. 

**Figure 5:**
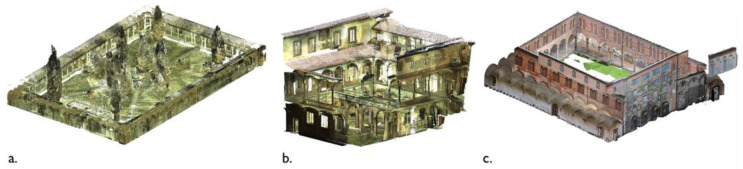
Point clouds for the three case studies considered: the Grand cloister of the Pisa Charterhouse (**a**); the Grand-Ducal cloister of the Pisa Charterhouse (**b**); the cloister of the National Museum of San Matteo in Pisa (**c**).

The case studies relate to the typology of medieval cloisters: a central area is closed on its perimeter by recurring architectural elements such as columns, moldings, arches and vaults that form a series of open galleries (Figure 5). In detail:

- The Grand (or main) cloister of the Pisa Charterhouse. The cloister, extending an area of about 70 × 45 m, was built starting from the year 1375 and underwent major renovations in the 17th century. The perimeter walkway, covered by vaulted ceilings and enclosed by marble columns, once provided access to the cells of the Carthusian fathers. The point cloud is the result of a Leica ScanStation C10 laser scanner survey (~10 M points).
- The Grand-Ducal cloister of the Pisa Charterhouse. Extending a rectangular-shaped area of 12 × 14 m, this cloister dates back to the 14th century. Its structure underwent several transformations around the 17th century, that lent it its current layout. The courtyard, with a central cistern, is overlooked by vaulted galleries; the two opposite sides of the cloister are connected, on the first floor, by an overhead walkway. The considered point cloud is the outcome of an integration between laser scanning and drone-based photogrammetric surveys (~6 M points).
- The cloister of the convent of San Matteo in Pisa. This cloister is located in the medieval convent of San Matteo in Pisa, which currently houses a National Museum. Major changes of its layout, dated to the 16th century, involved the construction of a *portico*, with granite columns closing the central space, Gothic windows and a cross-vaulted ambulatory. The survey was carried out via terrestrial photogrammetry and the resulting point cloud consists of ~12 M points.

The considered point clouds have different densities, but they are all set to a minimum space between points of 0.01 m.

### 3.2. Methods

The proposed methodological approach for Scan-to-BIM classification and reconstruction is divided into two macro-parts: in the first one, a supervised ML algorithm, the RF, is exploited to annotate the architectural components of a building from a point cloud survey. The workflow for this first phase was previously presented in reference [76], to which the reader could refer for further details and in-depth discussion on the ML-based data processing step. 

**Figure 6:**
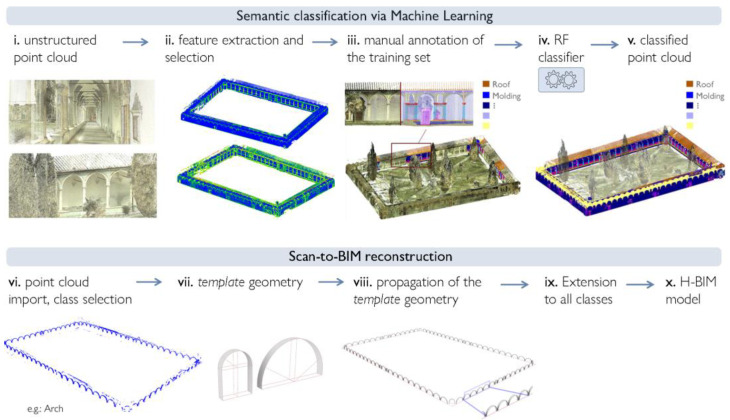
Two steps of the proposed approach: ML-based classification workflow, as illustrated in [76], and Scan-to-BIM reconstruction. The example provided refers to the case study of the Grand cloister of the Pisa Charterhouse.

The focus of this paper is rather on the second part of the workflow, concerning Scan-to-BIM reconstruction based on semantically annotated data. The latter takes place following the successive steps of: import of annotated data into the BIM environment; reconstruction and propagation, via VPL, of template geometries based on knowledge derived from historical treatises of architecture; and transfer of template geometries into BIM software as Autodesk Revit (Figure 6). The two steps of data segmentation and H-BIM reconstruction are both completed by a data validation process.

#### 3.2.1. Semantic Segmentation via ML

At first, semi-automated systems were exploited to properly interpret the 3D architectural scene from the input point cloud to improve the description and recognition of forms, materials, state of preservation. A ML-based segmentation procedure was used to assist the proper processing, management and semantic enrichment of digital heritage objects. The RF by Breiman [74] was used as the reference algorithm following the successful tests by Grilli [72]. This classifier is recognized as an effective tool for the classification of typological components of a building, outperforming other ML and DL approaches in terms of trade-off between training time, size of training data required and accuracy of the obtained results [26]. 

Starting from the extraction of appropriate covariance and radiometric features, and using a relevant set of training data, the RF is trained to classify, within digital models, the architectural elements that make up a historic building [76]. The procedure is broken down into five different steps:

(i) Neighborhood selection and feature extraction;
(ii) Feature selection;
(iii) Manual annotation on a reduced portion of the dataset (training set) to identify classes of elements;
(iv) Application of the RF classifier and consequent accuracy evaluation;
(v) Generation of an annotated 3D point cloud.

At first, a set of features is extracted in a chosen local neighborhood of each 3D point or image pixel (i). The choice of appropriate features and consequently, the choice of the local neighborhood in which they are computed, is fundamental in this phase as the predictive model is built to make predictions by recognizing the features that distinguish one class of elements from another. As the initial set of features may appear redundant or too large to be managed, the features are iteratively selected (ii). Readers can refer to previous work [76] for more details on the features’ description, extraction and selection steps. Subsequently, classes of recurring architectural elements are identified and labeled on a training set. This input data is used to perform a multi-scale classification via the RF to iteratively select the most relevant features; the classification process is thus run by considering a subset of features each time. For this reason, steps (ii) and (iii) are strictly interrelated. For the RF, the number of trees, $N_{trees},$ is set to 100 and the hyperparameter optimization accounts for overfitting through a 10-fold cross validation procedure.

Upon its completion of training using the training set of annotated data, the predictive model is applied to the remaining part of the point cloud or image (non-manually annotated), so to semantically label the classes of typological elements in the rest of the dataset. The accuracy of the classifier is finally assessed (iv), based on the comparison between true and predicted values on a *validation* set, that consists of almost the 25% of the labeled data (not used in the training phase). The performance evaluation is sorted out in the form of a *confusion matrix*, providing a measure of the number of correct and incorrect predictions, class by class. The on-diagonal elements stand for the True Positive (TP) values (correctly classified instances of the dataset), while the off-diagonal elements provide a measure of misclassifications: True Negatives (TN), False Positives (FP) and False Negatives (FN) values. The performance measures of Precision, Recall, Overall Accuracy and F-measure are derived from a combination of these values, as follows:

(1) $$Precision=\frac{TP}{TP+FP}$$

(2) $$Recall=\frac{TP}{TP+FN}$$

(3) $$Overall\;accuracy=\frac{TP+TN}{TP+TN+FP+FN}$$

(4) $$F-measure=2\cdot \frac{Recall\;\cdot Precision}{Recall+Precision}$$

The point cloud obtained at the end of this classification step is separated into its recurring architectural components: considering that the semantic structuring of heritage data could be beneficial in view of the construction of BIM-based representations, this classified point cloud is taken as initial data for the Scan-to-BIM reconstruction process. The hierarchical organization of the classes over the annotated point cloud reflects the logic of H-BIM environments, where each element (molding, vault, floor, wall, etc.) is defined by specific names, attributes and properties.

#### 3.2.2. Scan-to-BIM Reconstruction

Starting from the distinct classes of elements recognized on the point cloud through the segmentation process, so-called *template* geometries are reconstructed. These geometries are generated from the observation of architectural components that fall within a single point cloud class with reference to the architectural treatises. With VPL, a series of algorithms and rules are established for constructing the reference geometries of each type of component (*family*); subsequently, the reconstructed geometries are propagated to all elements belonging to the same typological class. 

A conceptual model is thereby reconstructed by treating and processing each class of architectural components separately; if carried out on the entire set of classes making up the architectural object, the replication of the *template* geometries yields a complete H-BIM information system. This way of classifying data appears consistent with the logic of the H-BIM process, whereby the model results from a combination of *smart* objects, properly differentiated in terms of type and morphology (e.g., roof, wall, floor, column, etc.) and grouped into *families* of architectural elements. In the overall model, each component is effectively discerned according to whether it belongs to one class or another. 

The model obtained at the end of the process, containing the 3D reconstruction of all typological element classes, can be used to construct H-BIM type representations, i.e., to build 3D archives of architectural heritage, which can be further enriched with information related to preservation and documentation.

In detail, for the reconstruction of *template* forms, reference geometries and proportions are derived, where available, from historical architectural treatises. Conceptual forms are hence generated for each class through the recognition and parametric reconstruction of related elementary parts, profiles and surfaces. The procedure is performed through VPL and is broken down class by class according to the following steps:

(i) Import of the annotated point cloud into 3D modeling environment, and extraction of the single class concerned by the reconstruction process;
(ii) Reconstruction of a *template geometry* for each class of architectural elements identified, while referring to architectural treatises and based on the definition of base construction plans, constraints, generating primitives, base profiles and ensuing functions of extrusion, loft, sweep, etc.;
(iii) Propagation of the template geometry to all elements belonging to a typological class, i.e., definition of element replica operations, so to enable the duplication of the defined geometry to multiple elements sharing same characteristics.

The mathematical and conceptual representation of each class, managed through generative modeling procedures, is entrusted to the creation of Non-Uniform Rational B-Splines (NURBS). 

Real-time generation, control, and editing of architectural forms is accomplished through the Grasshopper graphical algorithm editor, integrated in Rhino McNeel. Grasshopper, in particular, allows visual control of the 3D geometries reconstruction procedures by direct manipulation of nodes (algorithms) and wires. Finally, the Volvox (food4rhino.com/en/app/volvox, accessed on 18 December 2022) and Rhino.Inside.Revit (rhino3d.com/inside/revit/1.0/, accessed on 18 December 2022) plug-ins are used to connect the reconstructed 3D model with point cloud processing software and BIM platforms, respectively.

## 4. Results

### 4.1. Annotated 3D Data

**Figure 7:**
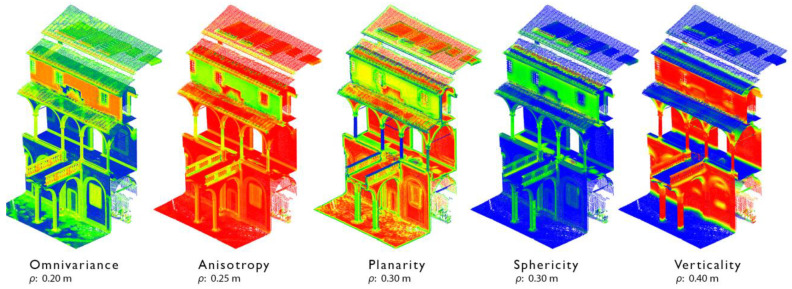
Many relevant features identified on a portion of the Grand-Ducal cloister dataset, Pisa Charterhouse, for different local neighborhood radii ρ.

**Figure A1:**
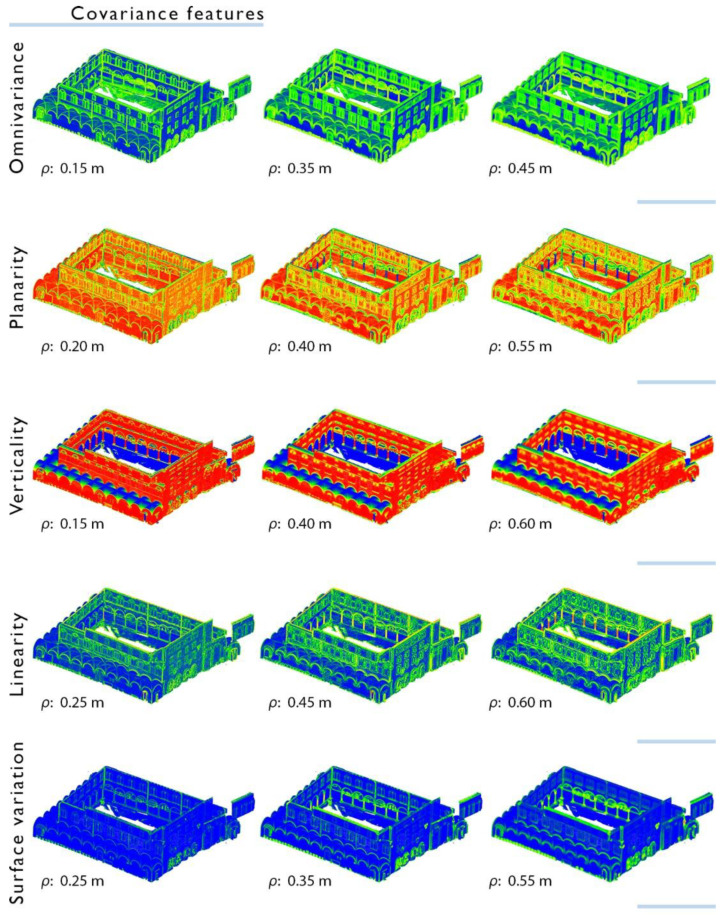
Examples of covariance features computed for different values of the local neighborhood radius using example from the Cloister of the National Museum of San Matteo.

**Figure A2:**
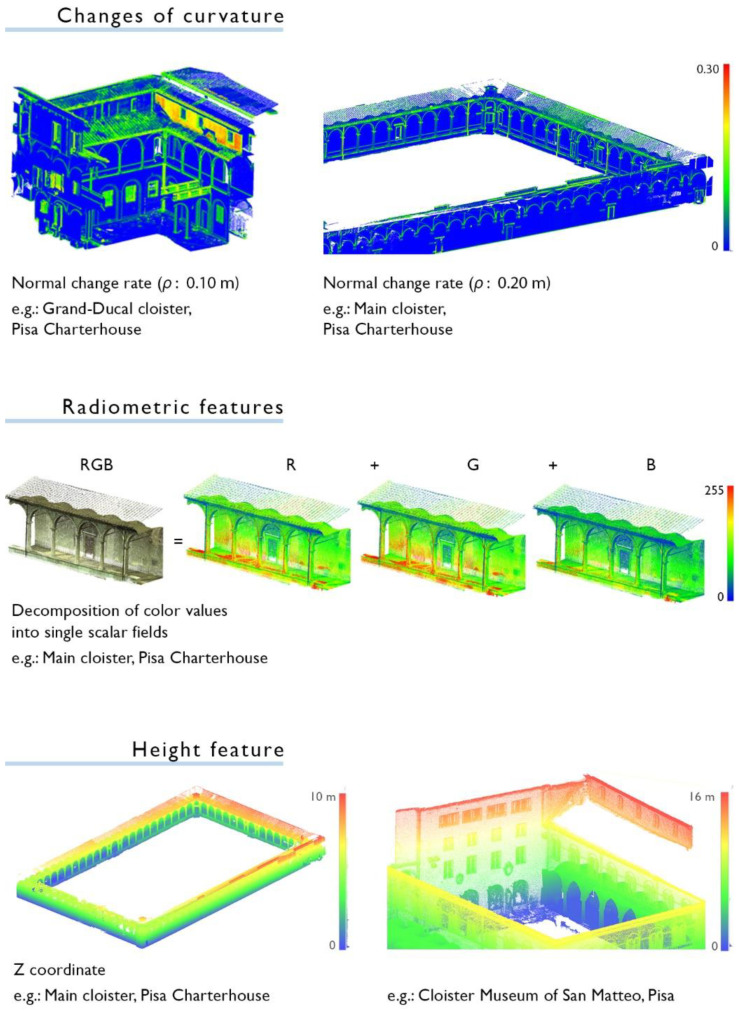
Examples of changes of curvature, radiometric features and height features computed for the different case studies.

At an initial stage, geometric features are extracted from input raw point clouds, with varying local neighborhood radii for each 3D point. Features, as covariance characteristics and changes of curvature, are computed and iteratively selected for each case study via a predictor importance estimate process [76] (Figure 7) in order to allow better distinction between one class and another. The choice of the local neighborhood in which features vary draws on considerations of the recurring dimensions of many elements composing the dataset, that are provided with a first estimate (e.g., the diameter of the columns, or thickness of certain architectural moldings, as well as other repetitive dimensions of the elements) so to extract geometric features considering selected ranges of the local neighborhood (e.g., ϱ = 0.2 m; ϱ = 0.4 m; ϱ = 0.6 m). Within the chosen ranges of ϱ, the covariance features are extracted from the covariance matrix, while the Normal Change Rate is extracted as a curvature measure describing for each point the speed of the orientation change [38]. Radiometric features, derived from the decomposition of the color space into single R, G and B scalar fields, and a height feature (the Z coordinate), are then considered as additional characteristics for each dataset. For additional samples of extracted covariance features -i.e., features depending on the distribution of 3D points in space (linearity, planarity, sphericity, omnivariance, etc.), colorimetric features and height features, the readers can refer to Appendix A (Figure A1 and Figure A2). 

**Figure 8:**
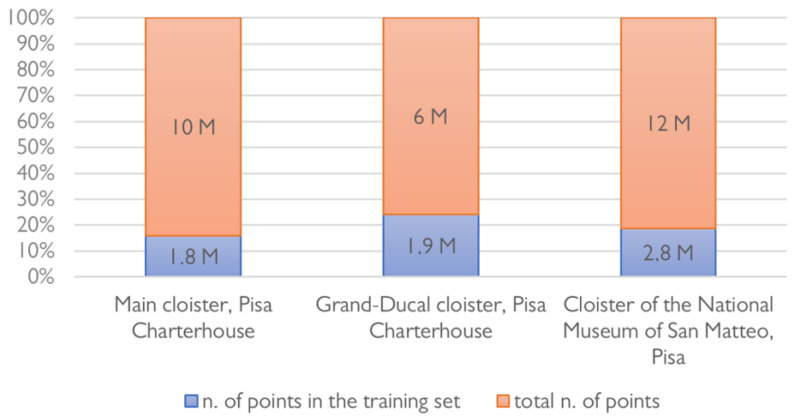
Percentage of training set used for learning over the three case studies considered.

**Figure 9:**
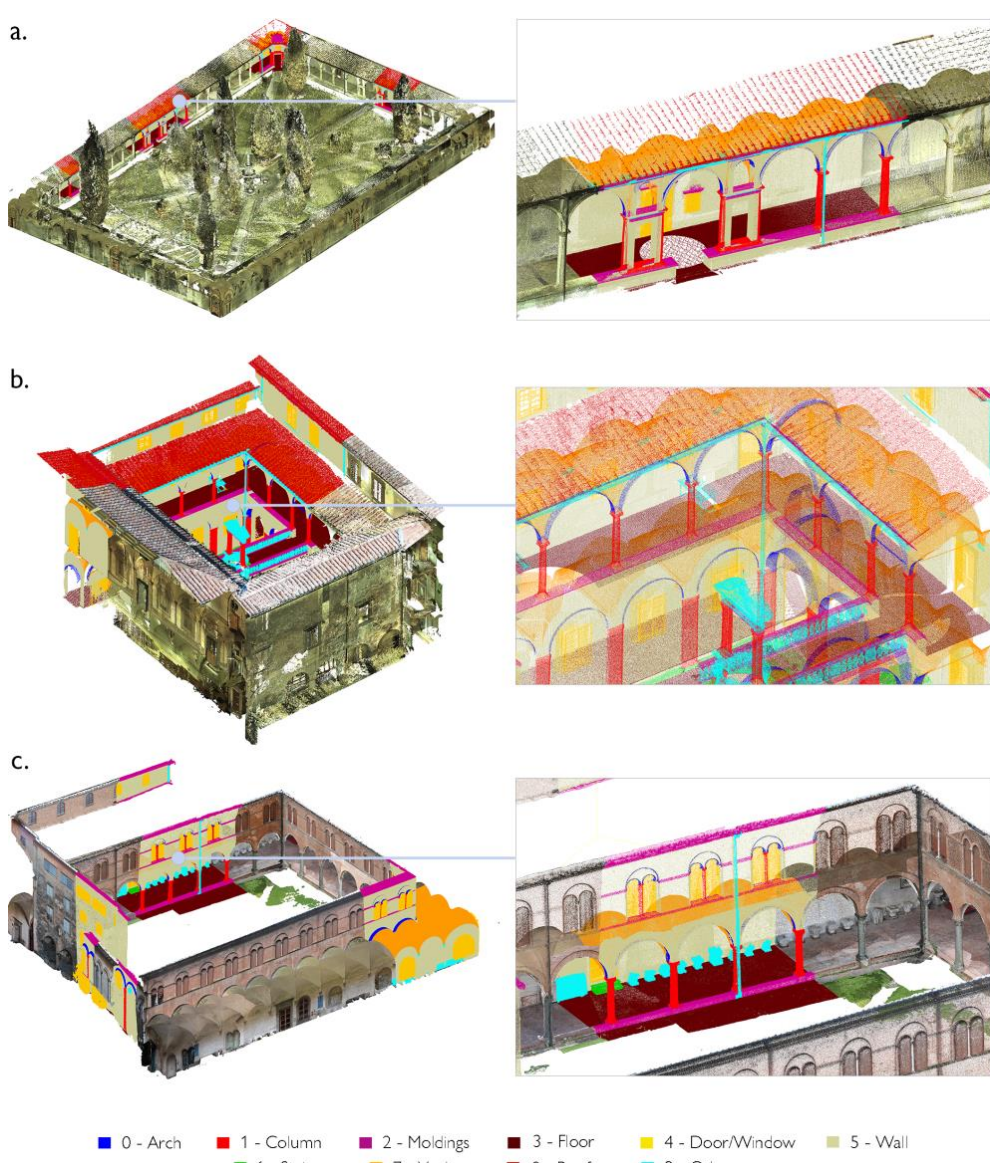
Training set for the three case studies. The Grand cloister (**a**) and Grand-Ducal cloister (**b**) of the Pisa Charterhouse; the cloister of the National Museum of San Matteo (**c**). The distinction of classes is conveyed visually, through a color legend.

After feature extraction, classes of architectural components are identified and annotated on a reduced portion of data samples, consisting of almost 20% of the total number of points of the point cloud (Figure 8). The so-called training set is specified each time for each case study; the point cloud is segmented based on the ten benchmark classes proposed by Matrone et al., 2020 [77] (Figure 9): arch, column, molding, floor, openings (door or window), wall, stair, vault, roof and other (all elements not belonging to the previous classes). 

The overall number of cases is 10 for the Grand-ducal cloister dataset, but it was reduced to 9 for the Grand cloister, as there is no ‘Class 6—Stair’, as well as for the San Matteo dataset, where ‘Class 8—Roof’ was not visible since the photogrammetric survey was ground-based and did not allow the description of the roofing structure. In any case, each identified class, for the three datasets, was associated with a specific label, to a class index varying from 0 to 10 and to a related color; the training set was chosen in a representative portion of the dataset, where all classes to be annotated are visible.

After the manual annotation of the training set, a first classification is run, via the RF algorithm, considering the multi-scale feature extraction (i.e., the whole set of features extracted at different local neighborhood radii). At the end of the learning process, an importance ranking can be displayed, showing the relevance score of each feature.

Starting from the importance ranking extracted, redundant and less relevant features are iteratively removed, and the RF is hence trained with a reduced subset of features. This step provides insight into the data, showing which features are more relevant to the classification task, thus reducing the dimensionality of the data to a set of almost 10–15 features and allowing the selection of a subset of predictors that adequately describe the identified classes. The feature ranking process is run each time for the three different case studies; surface variation, sphericity, anisotropy and verticality always appear among the most relevant features.

By comparing the results of the three different datasets in terms of feature selection, and by visually considering the variation of features along the datasets, many relevant considerations can be drawn up on the recurrence of some features: verticality, for instance, is more apt to the distinction between elements of the dataset that are mostly horizontal (floors, ground) or vertical (columns, walls), while omnivariance and sphericity enable the recognition of architectural moldings, arches, vaults and columns; anisotropy and planarity further support the classification of columns and the distinction of windows and doors from the wall. Moreover, they are valuable for depicting finer elements with horizontal development, e.g., the windowsill and the underroof moldings.

Normal change rate, as curvature feature, is appropriate to depict columns and arches, and to identify elements belonging to ‘Class 9—other’, as drain spouts. 

**Figure 10:**
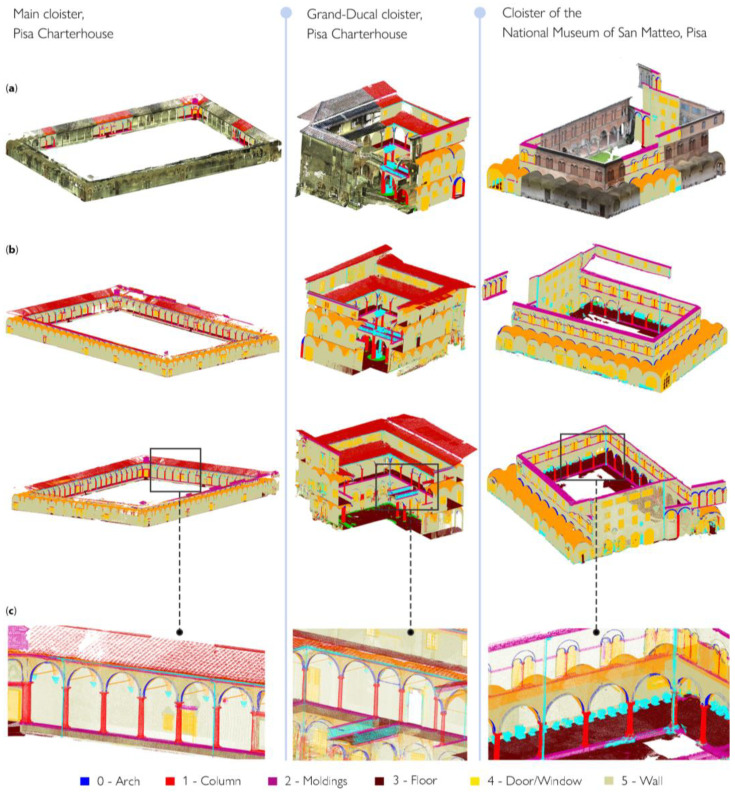
Training set (**a**) and segmentation results (**b**,**c**) for the three case studies.

**Figure 11:**
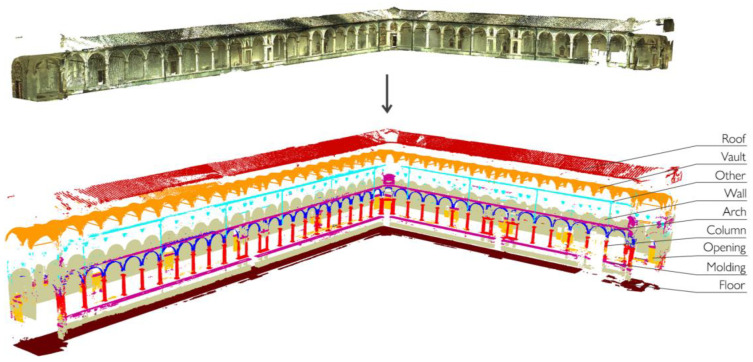
Semantic segmentation: exploded view of the classified components using example from the Grand cloister, Pisa Charterhouse.

The selected features associated with the manually annotated training set allow the RF classifier to be trained to extend classification to the entire point cloud. The procedure is followed for each of the cases studied and leads to the semantic segmentation results shown in Figure 10. Once classified, the point clouds can be differentiated according to the distinction of the architectural elements that compose them. As an example, Figure 11 provides a zoomed view of the several classes of recurring architectural elements that were recognized on a portion of the main cloister of the Pisa Charterhouse.

**Figure A3:**
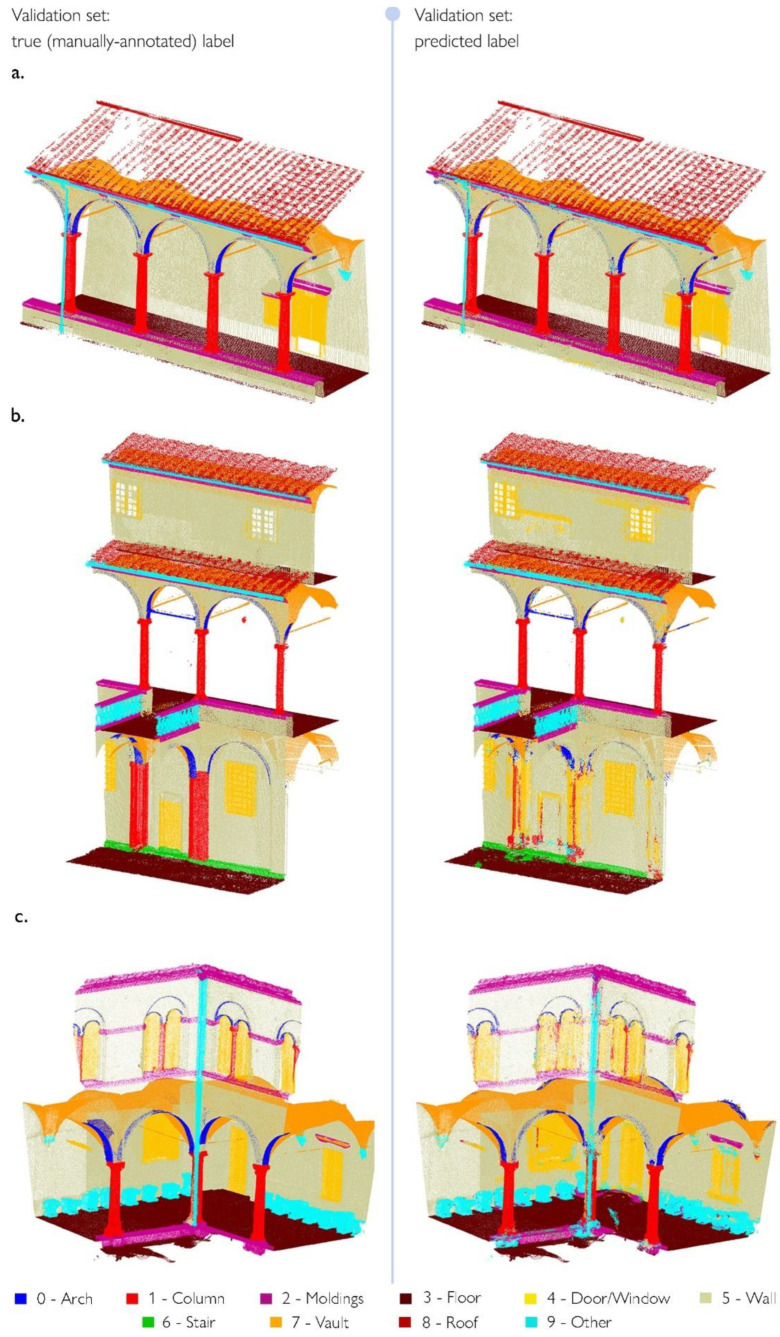
Validation sets. Comparison between true and predicted labels. The Grand cloister (**a**) and Grand-Ducal cloister (**b**) of the Pisa Charterhouse; the cloister of the National Museum of San Matteo (**c**).

**Figure A4:**
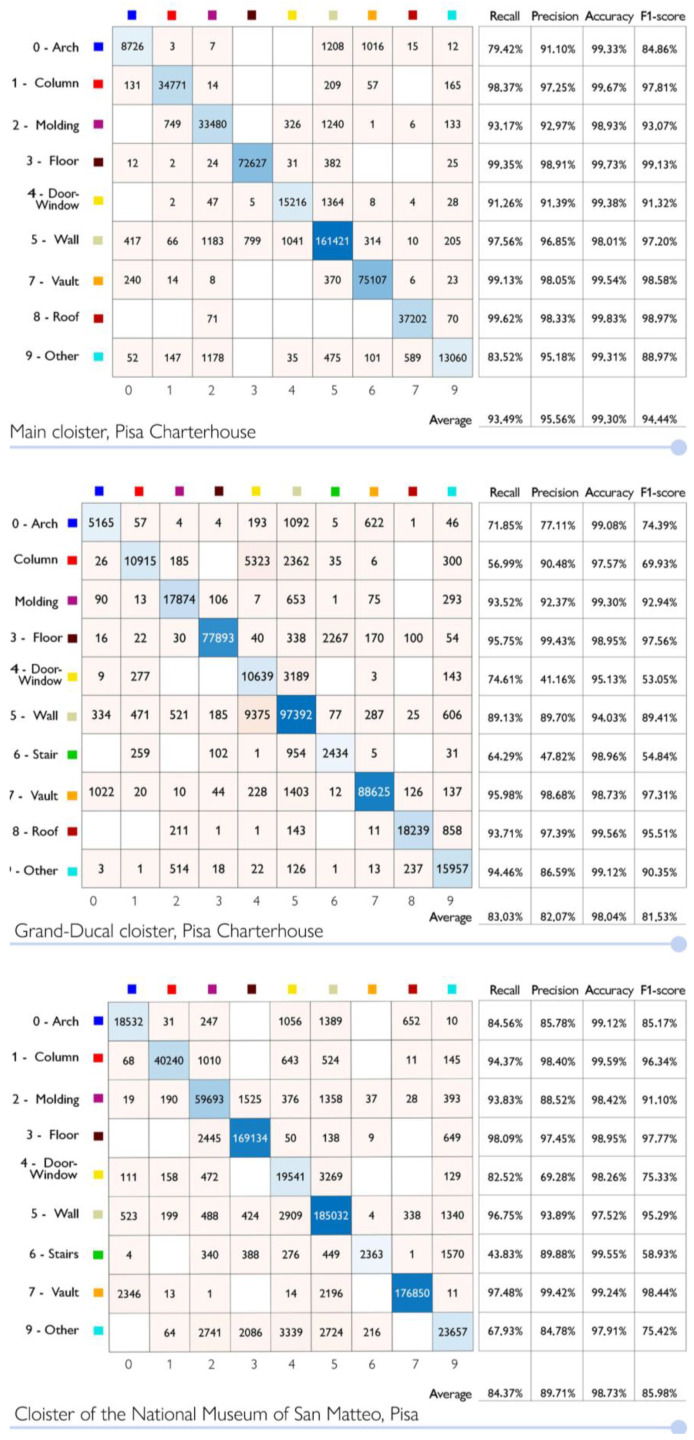
Confusion matrix and performance scores for the three case studies.

In order to evaluate the classifier performance, a validation set was considered, consisting of almost 25% of annotated portion of the data that was not previously used for training. On such validation set, the correspondence between the manually annotated labels and the predicted ones was tested by referring to the values of the confusion matrix and to the performance measures: Precision (1), Recall (2), Overall accuracy (3) and F-measure (4). The average values of the performance measures obtained for the three case studies are summarized in Table 1; a detailed description of the validation sets, confusion matrices and performance measures for the three cases is provided in Appendix A, Figure A3 and Figure A4.

### 4.2. Semantic-Based Reconstruction on Annotated Data

Since geometry-based approaches relying on supervised ML support the distinction of recurring typological elements as walls, columns, vaults, etc. on raw point clouds, the semantically segmented data is used here as a reference for the reconstruction of the HBIM models.

#### 4.2.1. Import of Annotated Data

**Figure 12:**
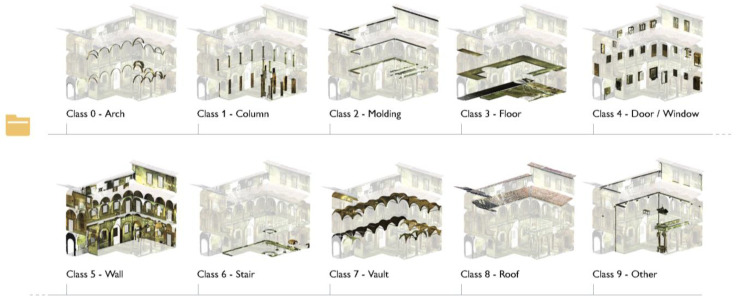
Two isolated classes of architectural components from the initial point cloud using example from the Grand-Ducal cloister, Pisa Charterhouse.

In the scan-to-BIM reconstruction process, the need first arises to preserve the classified 3D data even though the point cloud was imported into a 3D modeling environment. Through the semantic segmentation procedure, each architectural element identified within the point cloud can be isolated and shown individually (as in the example in Figure 12). This allows class information to be made available and visible in the transition to the 3D modelling environment, so that so-called template geometries can be reconstructed by acting on the manipulation and display of individual object classes over the point cloud between the point cloud and the Rhinoceros modeling environments.

**Figure 13:**
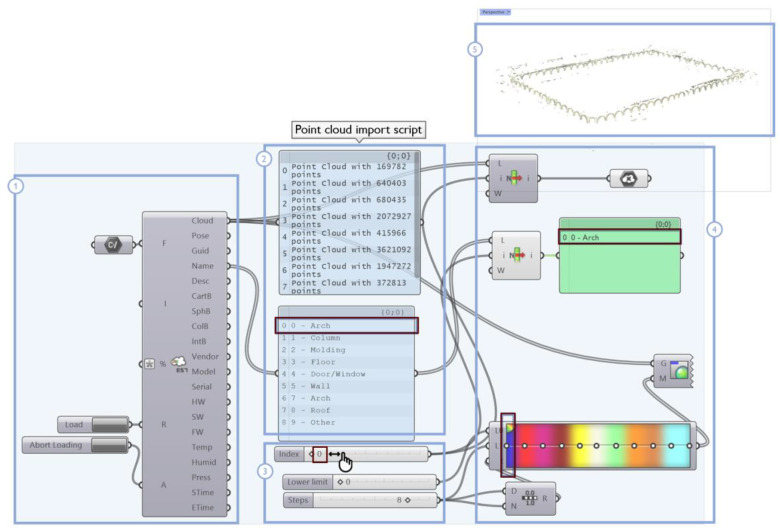
Point cloud import script. The meaning of each overlay rectangle is specified in the text using example Class 0—Arch, Grand cloister, Pisa Charterhouse.

**Figure 14:**
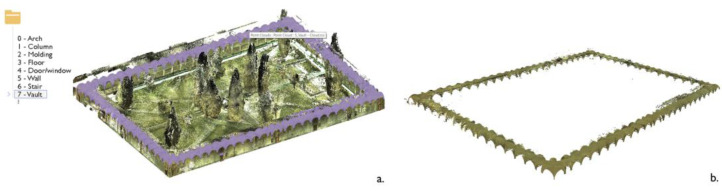
Segmented point cloud import. Selection (**a**) and isolation (**b**) of the ‘Vault’ class using example from the Grand cloister, Pisa Charterhouse.

In detail, the developed algorithm (Figure 13) reads and sorts the point cloud file (1), recognizing indices and colors that are associated with each class of typological elements. The original point cloud is segmented into multiple point clouds, each containing the points belonging to the individual class of architectural elements (2); then, the management of a special slider allows the user to directly select the index of a desired class (3). The colors, names and points associated with the selected class are selected (4) and the corresponding point cloud is displayed in the graphical user interface (5). The advantage of this procedure lies in allowing the user to activate or deactivate the display of a given class, depending on case-specific needs: only the 3D points belonging to the selected class are visible, while the remaining 3D points are hidden (Figure 14). 

This direct manipulation of architectural component classes on the original point cloud simplifies the modeling phase that is already in its first stage since the 3D reconstruction work can be carried out on an already segmented (reduced) dataset.

#### 4.2.2. Libraries of Template Geometries

Once the import of the semantically segmented point cloud into the modeling environment is completed, each class can be reconstructed in the form of a template geometry, i.e., a parametric 3D object that reproduces a particular recurring typological element (class or family of parametric elements). Again, the need to reconstruct geometries with modifiable and adaptable parameters leads to the choice of VPL, to model and edit the model by means of a series of rules and graphical processing operations (e.g., through the connection of nodes and wires in the VPL of Rhino Grasshopper). The following operations are performed via VPL algorithms:

- Definition of *template* conceptual shapes of each class of architectural elements identified on the point cloud, through a series of processing operations, rules (nodes) attributes and connections (wires).
- Control of the different graphic elements that compose the reconstructed shapes and direct manipulation of sliders related to their dimensions, extension and other and properties.

Many relevant classes of architectural types—such as the column, arches and vaults—are reconstructed with reference to architectural canons. For the case studies considered, Vincenzo Scamozzi’s treatise *L’idea dell’architettura universale* [78] is taken into account. This work inspired the renovation of many of the settings of the Charterhouse of Pisa in the 17th century. In detail, for the *template* shapes reconstruction process, the followed formalization approach is based on the work by De Luca et al. [53], consisting of three steps: interpretation of any knowledge referring to the shape; identification of the necessary modeling methods; and identification of relationships between elements.

With this approach, the elementary entities are identified and architecture primitives that constitute the basis of the representation method, are formalized. Once the 3D points belonging to each class of elements have been properly identified and shown on the 3D viewer, indeed:

- Reference building planes were detected and suitably oriented;
- A generating profile was created via VPL and, where necessary, a direction path was outlined.
- Functions such as revolution, sweep, extrusion, loft, etc. of the identified profile were used to build the targeted surface.

The parametric geometry constructed, representative of each class of elements, can be adapted over the point cloud through manipulation of sliders and parameters, and the related programming outputs are displayed in the Rhino3D graphical interface. For the formalization of *template* geometries, the moldings are studied first as they comprise the smallest and most trivial units of architectural elements corresponding to a semantic description of the building, and further combination of them yields more complex architectural elements. 

**Figure 15:**
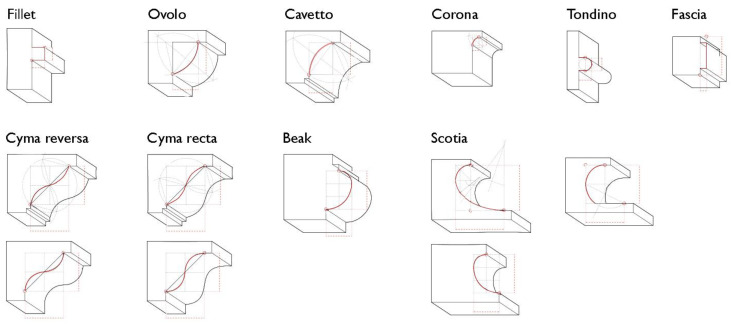
Study on template moldings for the formalization of their shape grammar.

**Figure 16:**
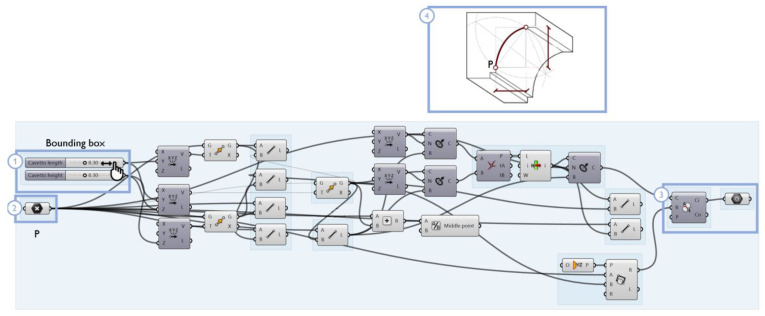
The cavetto molding can be modified by editing the sliders of the bounding box (**1**) and anchoring point P (**2**). The resulting curve is edited both on the VPL GUI (**3**) and in the 3D viewer (**4**).

**Figure 17:**
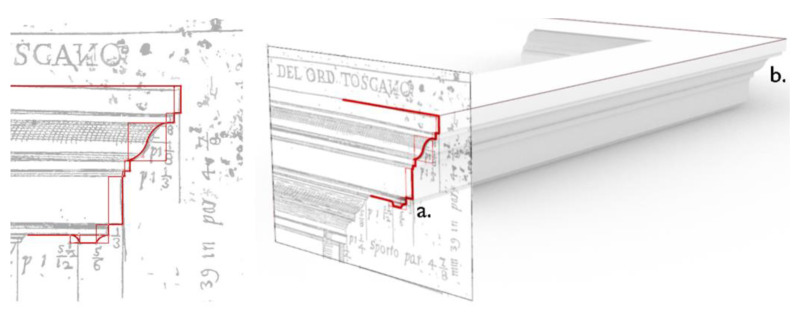
Generation of a profile for the combination of multiple moldings (to the left). The generating profile (**a**) is swept along the directing path (**b**) to create a 3D surface of the entablature (to the right).

Many moldings from Vincenzo Scamozzi’s treatise were analyzed and studied (Figure 15), and an example of the structure of their generation algorithms is shown in Figure 16: each molding is represented by a curve (drawn on a selected construction plane) and is included in a bounding box, i.e., a deformable section that defines the height and width of the element. A starting point and an ending point are the anchor points for any other moldings attached to the element. Considering the insertion points of the moldings as anchoring elements for the construction of subsequent moldings, more complex profiles are later provided (Figure 17). 

The application of extrusion, loft, sweep and revolution functions to the constructed generating profiles determines the *template* shape of a class.

**Figure 18:**
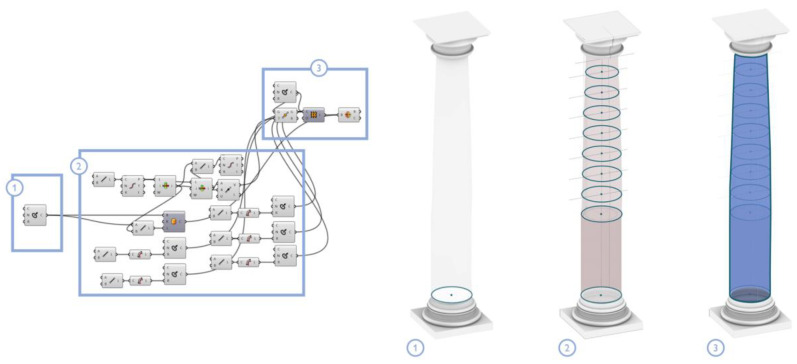
Creation of the column shaft: base circle (**1**), reference circles (**2**) and shape generation via the loft function (**3**).

Figure 18 shows the construction process of the ‘column shaft class’, for the case of the Grand Cloister dataset: first, descriptors and geometric attributes are defined for the construction of this architectural component. Then, the column shaft is built based on the study of the dimensional relationships between the diameter of the column base and its height, which allows for the establishment, at different heights, of reference circles. These circles are used to define, through a loft function, the conceptual shape of the concerned object, even representing the enthesis of the column shaft.

This approach can be extended to the entire set of classes, by a set of rules and visual scripting nodes that enable the construction of each architectural component.

#### 4.2.3. Information Propagation and Import into BIM Software

**Figure 19:**
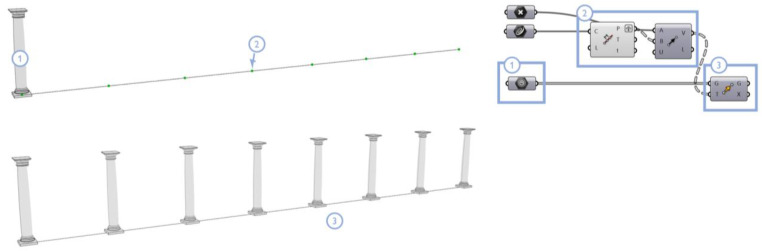
Example of propagation algorithm: template geometry (**1**); insertion points and *Move* node (**2**); and propagated geometries (**3**) using example from Class 1—Column, Grand cloister, Pisa Charterhouse.

**Figure 20:**
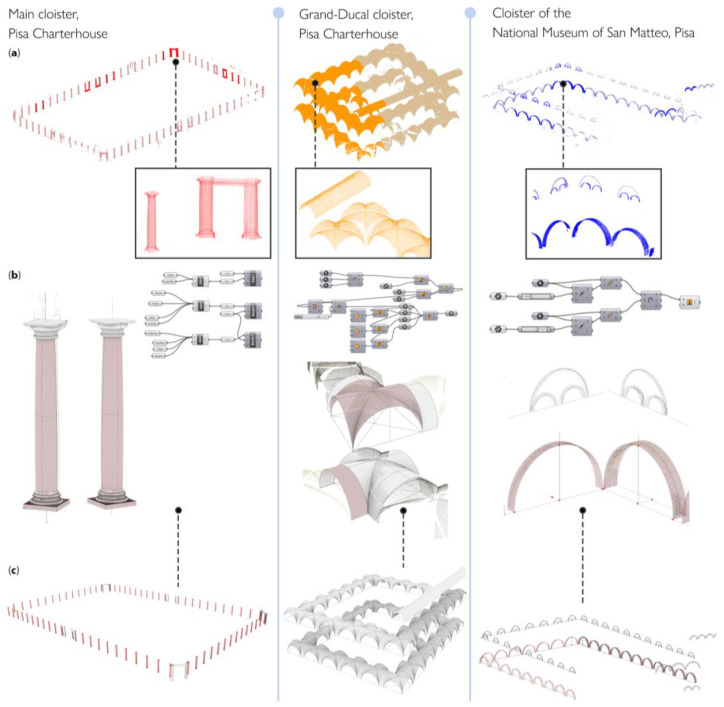
Examples of reconstruction of parametric components: original class (**a**), conceptual reference geometry (**b**) and propagation of the information to the whole class (**c**).

Once defined for each class, the geometry of the model is subsequently propagated to other parts of the point cloud, where the 3D points have been recognized as belonging to the same architectural type. The procedure is done through duplication and displacement nodes, particularly leveraging array, copy and translation operations (e.g., the *Move* node in Grasshopper). These operations allow the repetition, as many times as necessary, of the 3D geometry for each class of architectural components (Figure 19). Figure 20 shows the results obtained in the construction of some significant classes extracted from the considered datasets: the ‘column’ class for the main cloister, the ‘vault’ class for the Grand-Ducal cloister and the ‘arch’ class for the Museum of San Matteo. The creation and propagation of conceptual geometries by generative design rules allows the repetition and, where necessary, the modification of the parameter of these reconstructed geometries.

**Figure 21:**
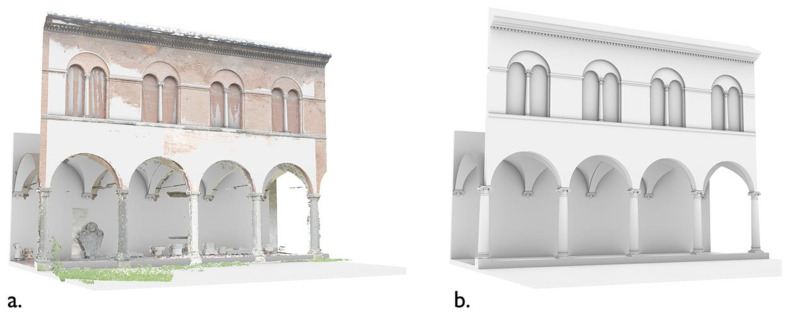
Resulting conceptual model for a portion of the cloister in the National Museum of San Matteo, with related point cloud (**a**,**b**).

**Figure 22:**
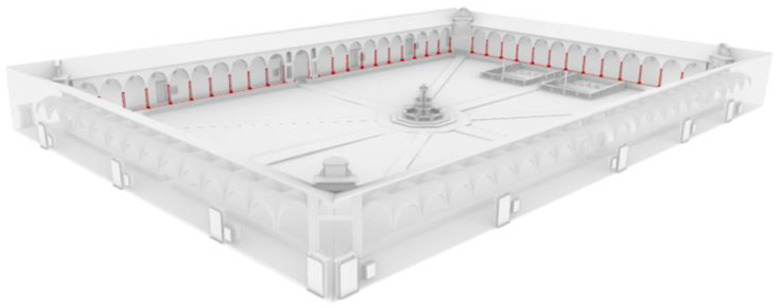
Resulting conceptual model for a portion of the main cloister in the Pisa Charterhouse and selection of the ‘Column’ class.

By extending the propagation of the *template* shapes to the set of all classes, a complete model of the whole building is finally achieved (Figure 21). Each architectural element identified within individual classes retains its own semantic description, as it is linked to the semantic decomposition of the architectural object and it is defined by a *template* shape (Figure 22).

Following these principles, each element is reconstructed independently of the software used and can be imported, for example, into BIM software, to be further enriched with non-geometric information (related, e.g., to materials, restoration and consolidation work, documentary and analytical sources, state of preservation, etc.).

**Figure 23:**
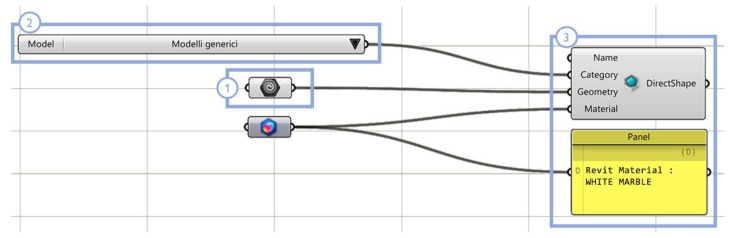
VPL import script via Rhino.Inside.Revit. The selected template geometry (**1**) is imported in Autodesk Revit as a generic model component (**2**), and it is associated with the Revit Material ‘white marble (**3**).

For instance, Grasshopper VPL could be linked to Autodesk Revit BIM software via the Rhino.Inside.Revit plug-in, as one possible way of importing the classified model in BIM environment: the algorithm displayed in Figure 23 allows the selection of architectural objects belonging to individual element classes and associates them with a Revit family, preserving their level of semantic description.

## 5. Discussion

The use of AI-based classification methods in common Scan-to-BIM processes empowers the automation of 3D model reconstruction from point clouds, in terms of time, raw surveying data management and semantic description. The key findings of this work may be identified following the breakdown into the two respective steps of semantic classification via ML (5.1) and BIM-based reconstruction (5.2).

### 5.1. Assessment of ML-Based Classification Methods

**Figure 24:**
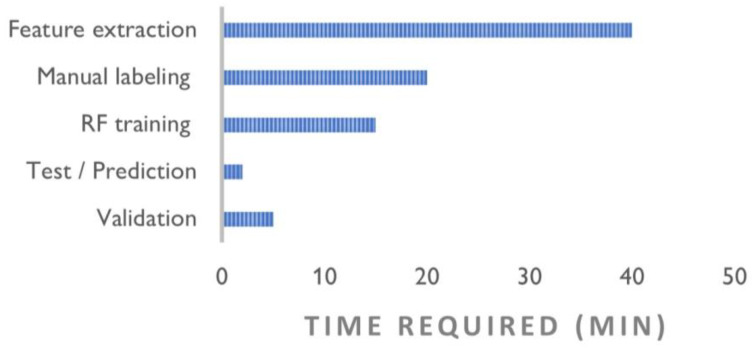
Average time required for the different phases of the classification process.

**Figure 25:**
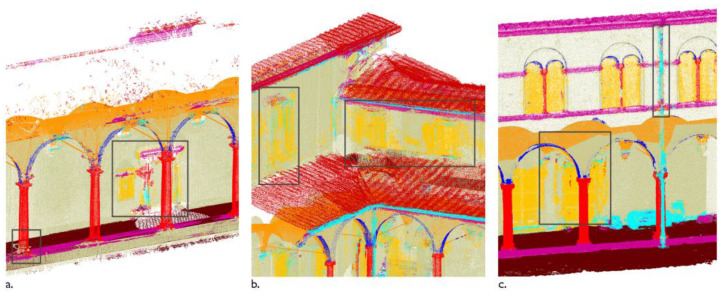
Misclassified elements in boundary regions. The Grand cloister (**a**) and Grand-Ducal cloister (**b**) of the Pisa Charterhouse; the cloister of the National Museum of San Matteo (**c**).

**Figure 26:**
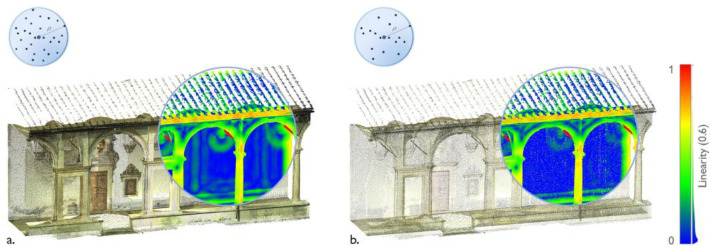
Example of a same feature (linearity, ϱ = 0.6 m) computed on two point clouds with higher (**a**) and lower (**b**) densities. Example from main cloister, Pisa Charterhouse.

Upon assessment of the performance scores of the three datasets being considered, the geometry-based classification returned an average accuracy of 98.73% and an F-score of 87.13%. The models accurately predicted the semantic segmentation results and significantly reduced the annotation time: Figure 24 summarizes the processing times for different stages of the workflow. However, some considerations need to be raised on the validation of this methodology:

- After observing the confusion matrices (Appendix A, Figure A4) and visually checking the data with the segmentation results, we observed misclassifications in the boundary regions, that is, in those areas that mark the boundary between one class and another. Specifically, as features are computed in a given local neighborhood ρ, feature extraction can be misleading for those 3D points that are in the boundary regions between classes (Figure 25). Those errors increase with increasing radius of the spherical neighborhood. This situation was mitigated, on the one hand, by adding discriminative radiometric features based on color information and, on the other hand, by choosing low (<0.6 m) values of ρ.
- Regions with similar developments (e.g., planar or cylindrical) in which geometric features may yield several values, can be misclassified as falling into the same class. For instance, the analysis of the off-diagonal elements of the confusion matrices suggested that Class 5—Wall’ and ’Class 4—Door and Window’ are often interchanged with each other, as both are characterized by predominantly planar behaviors.
- As the covariance and curvature features are computed in a given local neighborhood, the density of the point cloud influences the classification results. In other words, if two point clouds of a same object have different point densities, feature selection may produce two different results, as seen in the example in Figure 26. In order to align feature selection for different surveys of the same dataset, one could then plan to return the point clouds to the same density by means of a subsampling operation.

Besides these observations, it should be noted that, as the number of classes increased, greater similarity among them were found, and this implies that the number of semantic classes chosen can strongly influence the quality of the classification results; likewise, the amount of training data used can impact the resulting classification. In the case of laser scanning data, many sources of error, such as EDM centering, beam divergence and instrumental errors [79] which occurred during data collection, were not taken into account in the present study, although they could influence the classification results.

### 5.2. Assessment of the Scan-to-BIM Reconstruction Workflow

The separation of the semantic parts is the fundamental prelude to automation of the scan-to-BIM process and better correlation between the point cloud and parametric model. The semi-automated reconstruction of 3D mockups from survey data relies here on the visualization and import of the annotated data and on the subsequent construction and propagation of template geometries. With this approach, the geometric nature of the building components is reconstructed, the originally designed form is interpreted, and a reference geometry is identified and modeled for each class, following the logic of parametric BIM families. At the end of the process, visual programming techniques enable propagation and dimensional comparison of repetitive elements. A direct connection is in fact established between the point cloud and the reconstructed model on the level of individual classes of elements, and this avoids the loss or possible dispersion of information in the transition from the 3D survey to the parametric model. 

**Figure 27:**
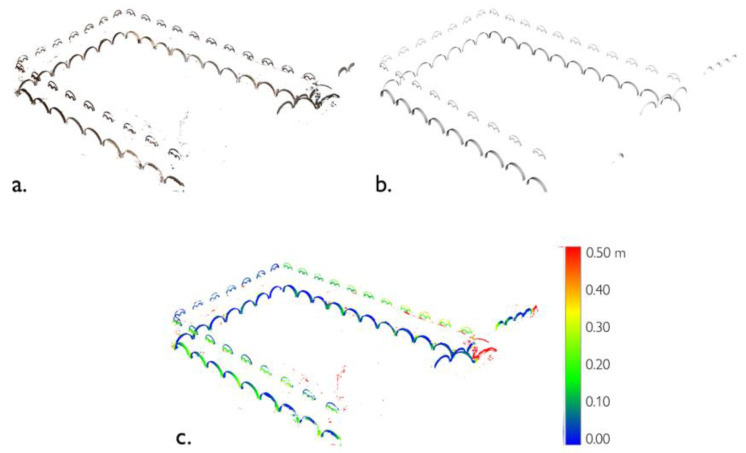
Real model (point cloud, (**a**)) and template model (**b**) are compared to extract a disparity map (**c**). The signed distances between the two models are expressed by a dedicated color scale., Example from Class 0—Arch, Grand-Ducal cloister, Pisa Charterhouse.

The resulting conceptual representation yields an effective support tool in the documentation of any architectural asset: the obtained digital model, being based on model geometries (with reference to architectural canons and treaties), becomes valid regardless of actual object changes and modifications, and additional information can then be inserted, retrieved, modified and updated within it, as a relevant basis for H-BIM-type systems. In fact, the use of architectural canons and the definition of a set of rules to reconstruct or modify a 3D object makes this system independent of the software used. This also implies that Grasshopper and Rhino3D do not represent the only environment in which such methods could be implemented, but rather other generative modeling software (such as Dynamo, implemented in Autodesk Revit software) could be exploited for the same task, if the same construction, modeling and operation processes are retrieved and repurposed accordingly. In addition, as a result of the connection drawn between the reconstructed classes and the semantic point cloud, it is pertinent to note that the two types of representations could be compared with each other, e.g., in terms of relative distances, in order to derive the extent to which, for each class of elements, a quasi-conceptual (digitally reconstructed) model deviates from the real data (e.g., from a point cloud acquired by survey). To this end, the comparison of the two (real and ideal) models could lead to the construction of disparity maps showing the variation, in space and time, of real (existing) architectural elements compared to the relative ideal model (Figure 27). For instance, this study could enable further refinement of the model geometry or could have an impact on the study of the evolution of an architectural style over time.

The conceptual model of the 3D connection point cloud allows each element belonging to a given class to be enriched with additional analytical or technical data. However, it is worth noting that the transfer of localized information—that is, information related to small portions of the model geometries—is not yet possible unless appropriate subdivisions of the reconstructed surfaces are provided to connect the information at a higher level of detail.

## 6. Conclusions

This work pointed at the automation of Scan-to-BIM workflows by combination of semantic segmentation methods exploiting AI and graphical algorithm editors for 3D modeling. 

At first, geometry-based classification approaches are exploited to enable, to different extents, the addition of a semantic label associated with the decomposition of the building into recurring architectural elements. ML algorithms, implemented by suitably leveraging manipulation, export and extraction of geometric and visual descriptors (features) from raw 2D or 3D data, significantly reduce the brute annotation phase, lessening the space for arbitrary and too subjective choices. However, user supervision, in terms of choice of the training set and decision on which classes should be used to partition the digital data, is crucial in determining the success of the classification and labeling process. In addition, high performance computing machines are required in the feature extraction and data-driven algorithm training phase. Moreover, mis-classification may occur in boundary regions, as well as in regions with similar development (geometry-based approaches) or with similar color characteristics or patterns (texture-based approaches). The generalization of the same ML algorithm to other datasets, pertaining to different architectural types and/or built in different periods, as well as the establishment of larger annotated datasets to train deep neural networks, are possible future developments in this domain.

The semantically segmented point cloud is later exploited for the construction of a reference model, composed of template geometries, following the logic of H-BIM type information systems. The proposed procedure enables the reconstruction of Heritage-Building Information Models starting from annotated 3D survey data. The reconstructed H-BIM model preserves the semantic link with the semantically annotated point cloud, at the level of the single classes of detected architectural components and can be leveraged for further enrichment with non-geometric (analytical, knowledge-related) information. However, it is noted that the annotation process, although being trivial when referred to the single architectural component, becomes tricky in cases of localized annotation.

Considering this aspect, future work could be focused on developing additional possibilities of semantic structuring and transfer of more localized information, relying on the definition of suitable tiling procedures of the template model geometries. These experiments could be aimed at noting, inter alia, the presence of frescoes and decorative parts, degradation phenomena, and crack patterns, repair and restoration interventions.

The integration of reconstructed H-BIM models and existing H-GIS systems, at the urban and territorial scale, could even be the subject of future research.

## Figures and Tables

**Table 1:** Average performance measures obtained for the classification of the three datasets.

	Grand Cloister, Pisa Charterhouse	Grand-Ducal Cloister, Pisa Charterhouse	Cloister Museum of San Matteo, Pisa
n. of classes	9	10	9
Avg. classes	93.49%	83.03%	84.37%
Avg. precision	95.56%	82.07%	89.71%
Avg. accuracy	99.30%	98.04%	98.73%
Avg. F1-score	94.44%	81.53%	85.98%

## Data Availability

The data presented in this study are available on request from the corresponding author.

## Abbreviations

AI	Artificial Intelligence
BIM	Building Information Modeling
DL	Deep Learning
FN	False Negatives
FP	False Positives
H-BIM	Heritage- or Historic- Building Information Modeling
ML	Machine Learning
RF	Random Forest
TN	True Negatives
TP	True Positives
VPL	Visual Programming Language
